# ^19^F NMR studies on γ-butyrobetaine hydroxylase provide mechanistic insights and suggest a dual inhibition mode†
†Electronic supplementary information (ESI) available. See DOI: 10.1039/c9cc06466d


**DOI:** 10.1039/c9cc06466d

**Published:** 2019-11-08

**Authors:** Robert K. Leśniak, Anna M. Rydzik, Jos J. A. G. Kamps, Amjad Kahn, Timothy D. W. Claridge, Christopher J. Schofield

**Affiliations:** a The Department of Chemistry , University of Oxford , 12 Mansfield Road , Oxford , OX1 3TA , UK . Email: tim.claridge@chem.ox.ac.uk ; Email: christopher.schofield@chem.ox.ac.uk

## Abstract

The final step in the biosynthesis of l-carnitine in humans is catalysed by the 2-oxoglutarate and ferrous iron dependent oxygenase, γ-butyrobetaine hydroxylase (BBOX). ^1^H and ^19^F NMR studies inform on the BBOX mechanism including by providing evidence for cooperativity between monomers in substrate/some inhibitor binding. The value of the ^19^F NMR methods is demonstrated by their use in the design of new BBOX inhibitors.

## Abstract 2

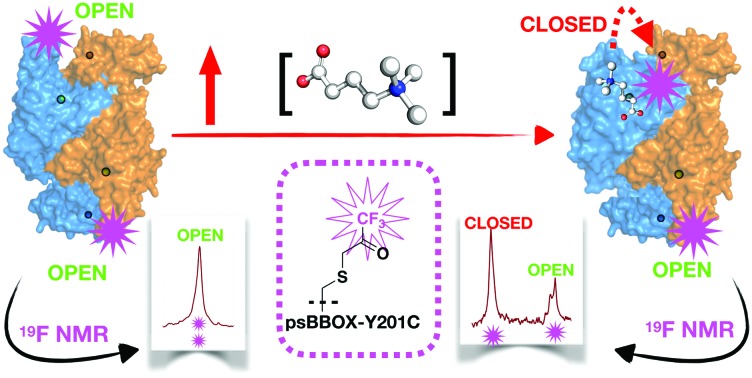
 ^19^F and ^1^H NMR studies on fluorine labelled γ-butyrobetaine hydroxylase provide mechanistic insight into substrate and ligand binding, suggesting cooperativity between two monomers.

## 

The final step in l-carnitine (**1**) biosynthesis in humans is catalysed by the 2-oxoglutarate (2OG, **2**) and ferrous iron dependent oxygenase, γ-butyrobetaine hydroxylase (BBOX) (Fig. 1A).^1^,^2^ Carnitine plays a crucial role in lipid metabolism by enabling long-chain fatty acid transport into mitochondria for β-oxidation.^3^,^4^ Approximately a quarter of total carnitine in humans is produced endogenously, with the remainder from alimentation, *e.g.* red meat.^5^,^6^ Excess carnitine is proposed as a cardiovascular disease risk, due to its gut metabolism to *N*-trimethylamine oxide.^7^ Carnitine is proposed to indirectly regulate carbohydrate metabolism *via* modulation of the acetyl-CoA/CoA ratio,^8^,^9^ which affects pyruvate dehydrogenase activity.^10^,^11^ Targeting carnitine biosynthesis to therapeutically regulate cellular energy metabolism to treat cardiovascular diseases is thus of interest.^12^,^13^

**Fig. 1:**
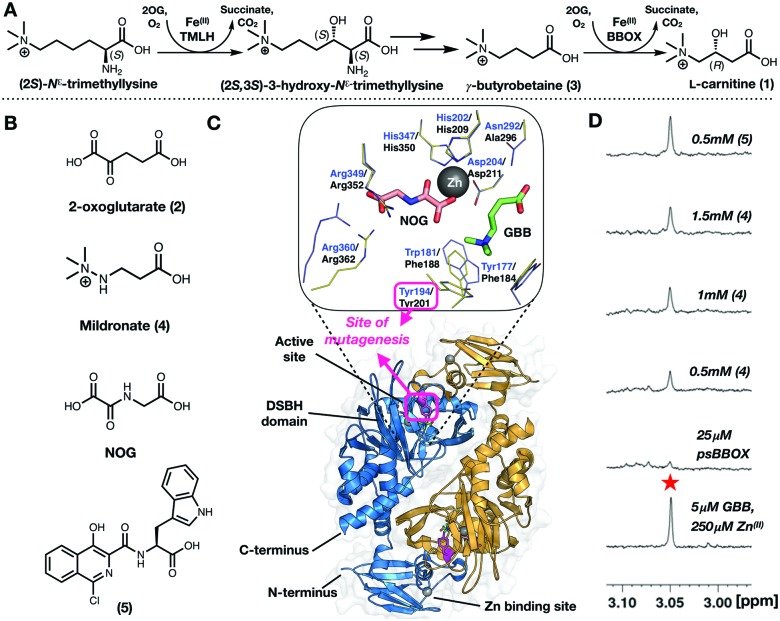
(A) Dimeric γ-butyrobetaine hydroxylase (BBOX) is a 2-oxoglutarate oxygenase. (B) BBOX inhibitors: Mildronate (**4**); isoquinoline (**5**). (C) Overlaid γ-butyrobetaine (GBB, **3**; green sticks) and *N*-oxalylglycine (NOG; salmon sticks) binding residues of hBBOX (blue/orange cartoon (dimer view)) blue lines (PDB: ; 3O2G)^1^ and a psBBOX model^23^ (yellow lines). psBBOX Tyr201 (Y194 hBBOX,^1^ pink sticks) is involved in GBB quaternary ammonium ion recognition;^24^ it is located in a ‘flexible-loop’ region. (D) Titrations of (**4**) manifest only ∼60% GBB displacement using a ^1^H NMR reporter assay;^25^ by contrast (**5**) apparently displaces ∼90% GBB.

Mildronate (**4**) (Meldonium, THP, Met-88) (Fig. 1B) is a clinically used cardioprotective agent,^14^ which has received attention given its proposed performance enhancing abilities leading to (ab)use in the sporting community.^15^,^16^ Definitive evidence for its effects, and the biological modes of action of Mildronate, are lacking; it is proposed to cause a change in metabolism from mitochondrial fatty acid β-oxidation towards peroxisomal metabolism and glycolysis, *via* reduction of carnitine levels, due to inhibition of BBOX and of carnitine uptake.^9^

Carnitine is used as a fat-loss aid^17^,^18^ and to treat cardiovascular conditions and carnitine deficiency.^19^,^20^ Hence, there is interest in carnitine fermentation. Carnitine is biosynthesized by microbes, including *Pseudomonas* spp (*e.g.* sp. AK1) producing a human BBOX homologue.^21^,^22^ Like human BBOX (hBBOX, sequence similarity: ∼30%) *Pseudomonas* sp. AK1 BBOX (psBBOX) is a 2OG and Fe^(II)^ using oxygenase.^21^

Crystallography reveals BBOX to be dimeric; each monomer containing a 2OG oxygenase characteristic double-stranded β-helix fold and typical Fe^(II)^ and (co)substrate binding elements (Fig. 1C).^1^ Recombinant psBBOX is produced efficiently in *Escherichia coli* (80 mg L^–1^);^23^ by contrast, recombinant hBBOX is more difficult to produce in bacteria. Given its high yield, recombinant psBBOX is a useful model enzyme for studying BBOX and related enzymes, like trimethyllysine hydroxylase.^23^

NMR based reporter BBOX assays, using either Zn^(II)^ or Mn^(II)^ (making use of paramagnetic relaxation enhancement (PRE)) to observe ligand binding are reported.^25^ These enable monitoring of co/substrate 2OG/γ-butyrobetaine (GBB; **3**) psBBOX binding and inform on binding modes of inhibitors, including whether they displace GBB and/or 2OG. We now report ^19^F NMR studies on ligand binding to BBOX; the work was initiated following ^1^H NMR observations concerning the binding of Mildronate to psBBOX. The results inform on the BBOX mechanism by revealing cooperativity between monomers during substrate binding. The value of the ^19^F NMR methods is demonstrated by their use in identification of new BBOX inhibitors.

During ^1^H ligand observed studies on the binding of Mildronate to psBBOX, we observed that attempted displacement of GBB by Mildronate from the psBBOX-Zn^(II)^-2OG–GBB complex (and *vice versa*) does not proceed to more than ∼60%, *i.e.* to give an apparent ∼1 : 1, Mildronate : GBB complex (Fig. 1D). Mildronate is a close structural analogue of GBB, that under catalytic conditions is a competitive hBBOX substrate undergoing fragmentation *via* Stevens type rearrangement to give several products.^26^ By contrast with the Mildronate results, on titration of the 2OG-competing and metal-chelating BBOX inhibitor (**5**) (IC_50_ = 0.9 μM)^25^ near complete GBB displacement was observed (Fig. 1D). Since psBBOX is dimeric^21^,^22^ (Fig. 1C), these observations led to the proposal that binding of a second molecule of GBB (or Mildronate) to the psBBOX-Zn^(II)^-2OG–GBB complex strengthens binding of the first GBB molecule, *i.e.* there is cooperativity in substrate binding between the monomers of the dimer.

We proposed further insights into the apparent cooperative ligand binding could be achieved using protein-observed fluorine (PrOF) NMR spectroscopy.^27^^19^F NMR was chosen over traditional methods using ^15^N/^13^C labelling due to the near 100% natural abundance of ^19^F,^28^ the high ^19^F signal sensitivity (83% relative to ^1^H),^27^ and, importantly for BBOX studies, the ease with which one can produce ^19^F labelled proteins and interpret spectra of large macromolecules.

Given a lack of crystallographic data for psBBOX, choice of the position for ^19^F labelling was based on hBBOX crystallography.^1^,^29^ psBBOX Tyr201 (Tyr194, hBBOX) is located on a ‘flexible-loop’ which plays a role in catalysis *via* recognition of the GBB quaternary ammonium group (Fig. 1C).^24^ To study psBBOX using ^19^F NMR *via* use of the thiol-selective reagent 3-bromo-1,1,1-trifluoroacetone (BTFA), site-specific cysteine substitution of psBBOX at Tyr201 was carried out (Fig. 2A).^30^ Treatment of wt-psBBOX with BTFA manifested no labelled product by MS (Fig. S1 and S2, ESI†); by contrast Y201C was efficiently labelled. BTFA was apparently selective for Cys201, independent of incubation time and equivalents of BTFA used, despite the presence of other cysteines in psBBOX. These results support the proposed solvent exposed nature of Tyr/Cys201 (at least in uncomplexed psBBOX) and are consistent with the proposed dynamic nature of the ‘flexible-loop’ region (Fig. 2).^1^

**Fig. 2:**
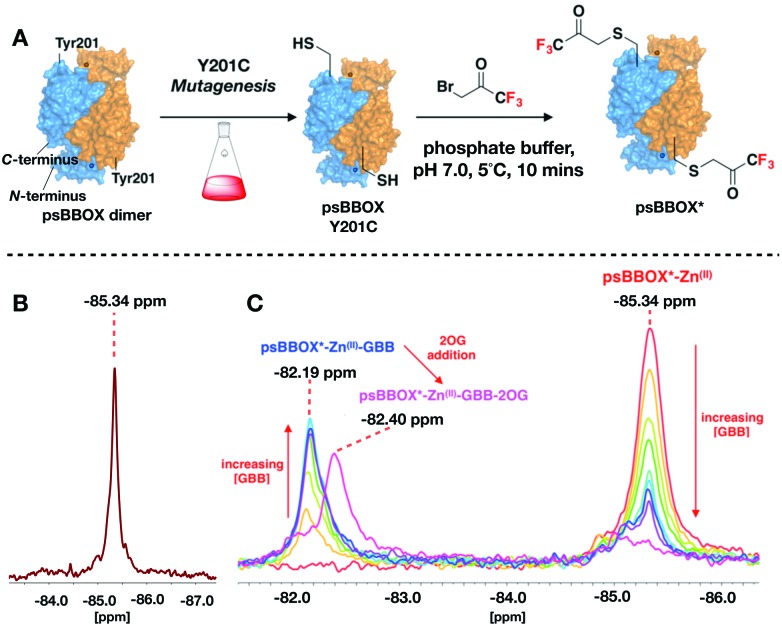
(A) Labelling of the Y201C psBBOX (blue/orange surface) using 3-bromo-1,1,1-trifluoroacetone (BTFA) to give psBBOX*. (B) ^19^F NMR spectra of apo-psBBOX*. (C) ^19^F NMR spectra obtained from titrations of psBBOX*-Zn^(II)^ with GBB/2OG. (See ESI† for details).

BTFA labelled psBBOX-Y201C (psBBOX*) was catalytically active by ^1^H NMR (*V*_max_ = 2.6 μM s^–1^, *k*_cat_ = 1.4 s^–1^, *K*_m_ = 362 μM, Fig. S3–S5, ESI†), though less so than wt-psBBOX (*V*_max_ = 1.3 μM s^–1^, *k*_cat_ = 5.2 s^–1^, *K*_m_ = 696 μM, Fig. S5, ESI†). A sharp singlet was observed with psBBOX* by ^19^F NMR at –85.34 ppm relative to TFA (Fig. 2B), indicating that the flexible loop of dimeric psBBOX* exists as a single distinct symmetrical conformer in solution and/or that flexible loop movement is fast on the NMR timescale, such that a time averaged shift is observed.

To monitor psBBOX* ligand binding without turnover, we used Differential Scanning Fluorimetry thermal shift studies with wt-psBBOX to identify an Fe^(II)^ surrogate: Ni^(II)^ and Zn^(II)^ were identified as candidates (Fig. S6, ESI†). Initial ^19^F NMR experiments with psBBOX* showed Zn^(II)^ enabled visualisation of co/substrate binding events (Fig. 2C); the results with Ni^(II)^ were more complex (Fig. S7, ESI†). Titration of GBB with psBBOX*-Zn^(II)^ manifested a second signal at –82.19 ppm which increased in intensity with increasing GBB concentration (Fig. 2C). Thus, Zn^(II)^ was used in subsequent ^19^F NMR psBBOX* ligand binding studies.

The chemical shift change (Δ*δ*_F_) of 3.15 ppm, relative to the psBBOX* signal at –85.34 ppm, observed on GBB addition to psBBOX*-Zn^(II)^ indicates a significant change in local environment for the ^19^F nucleus, consistent with the labelling position being close to the GBB trimethylammonium binding site (Fig. S8, ESI†). By contrast to GBB, titration of 2OG with psBBOX*-Zn^(II)^ complex did not yield a second signal, but manifested broadening and a slight shift of the ^19^F resonance, in a 2OG concentration dependent manner (Fig. S8, ESI†). This type of observation is typically observed in PrOF NMR with weak binding ligands that exhibit equilibrium binding kinetics in an intermediate exchange regime. These results are consistent with the unusually high *K*_m_ of wt-psBBOX for 2OG (532 μM).^23^

Addition of 2OG to the psBBOX*-Zn^(II)^-GBB complex manifested a shift of the signal at –82.19 ppm to –82.40 ppm (Fig. 2C), likely representing a state in which all co/substrates are bound. Attenuation of the putative psBBOX* signal at –85.34 ppm was also observed, suggesting 2OG addition promotes GBB binding. The combined effects of 2OG on the psBBOX*-Zn^(II)^-GBB support the results obtained with the ^1^H NMR reporter assay, *i.e.* GBB has a relatively high binding affinity for psBBOX in the presence of 2OG (*K*_D_ = 5 μM).^25^ They are also consistent with the proposed cooperativity on binding of a second GBB molecule being due to enhancement of binding of the first, *via* promotion of closure of the flexible loop (Fig. 3A). A comparable binding affinity was observed for GBB with psBBOX* (*K*_D_ = 8.9 μM, Fig. S9, ESI†).

**Fig. 3:**
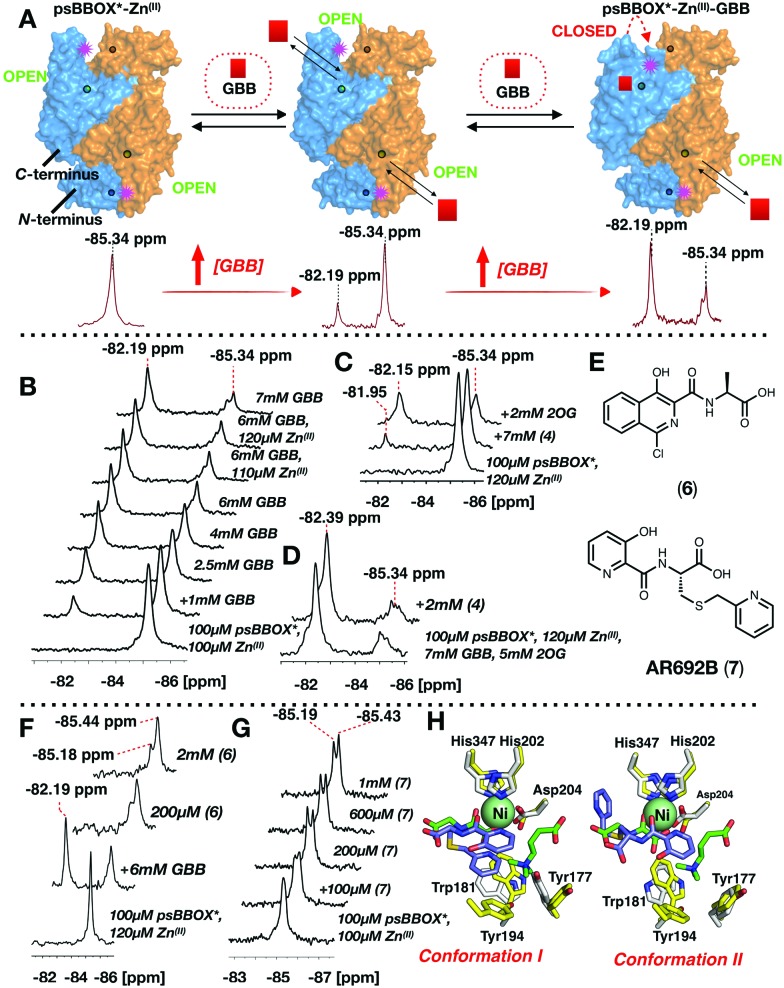
^19^F Labelled psBBOX* can be used to monitor ligand binding. (A) We propose binding of GBB to a second monomer strengthens binding of GBB to the first monomer, *via* a conformational change. Site of BTFA labelling: pink stars. psBBOX* monomers: blue or orange surfaces. Circles: C- and N-terminus Zn^(II)^ binding sites. (B) ^19^F NMR of titrations of GBB with psBBOX*. (C) Evidence 2OG influences Mildronate (**4**) binding to psBBOX*. (D) Mildronate does not change the ^19^F NMR spectra observed with psBBOX*-Zn^(II)^-GBB–2OG under the tested conditions. (E) hBBOX inhibitors (**6**) and (**7**). (F) Addition of (**6**) attenuates the GBB binding signal. (G) ^19^F NMR spectra of titrations of AR692B (**7**) with psBBOX* may reflect two crystallographically observed binding modes. (H) The two conformations of (**7**) with hBBOX (PDB: ; 4C8R). Overlays of the structures of GBB (green sticks), and NOG (green sticks) at the hBBOX active site (yellow sticks), and (**7**) (blue sticks) bound to hBBOX (white sticks) are shown.

Titration of Mildronate with psBBOX*-Zn^(II)^ manifested only low levels of a comparable second signal, so contrasting with GBB titrations; high Mildronate concentrations were required (∼70 : 1, Mildronate : psBBOX* Zn^(II)^) to observe binding (Fig. 3C). Notably, addition of 2OG to the psBBOX*-Zn^(II)^–Mildronate complex clearly gave a second signal at –82.15 ppm, with a similar Δ*δ*_F_ to that observed on addition of 2OG to the psBBOX*-Zn^(II)^-GBB complex (Δ*δ*_F_ 3.19 ppm, Fig. 2C and 3C, *K*_D_ = 12.4 μM, Fig. S10, ESI†). We were unable to detect inhibition of psBBOX* catalysed GBB turnover by Mildronate by ^1^H NMR (Fig. S10, ESI†). Addition of Mildronate to psBBOX*-Zn^(II)^–GBB-2OG, did not manifest detectable changes using ^19^F NMR (Fig. 3D). These results agree with the reported relatively weak affinity of Mildronate for hBBOX (IC_50_ = 34–60 μM).^29^,^31^

Studies with different types of reported BBOX inhibitors^25^,^32^ (Fig. S11, ESI†) and substrate/product analogues (Fig. S12, ESI†), further reveal utility of the ^19^F NMR method for monitoring subtle differences in ligand binding modes for even closely related compounds, *e.g.* for 2OG and its close analogue *N*-oxalylglycine (NOG) (Fig. S13 and S14, ESI†) and for compounds in the same series (Fig. S11, ESI†).

Titration of a potent psBBOX inhibitor (**6**) (IC_50_ = 0.2 μM^25^) with psBBOX*-Zn^(II)^ manifested signals at –85.18 ppm and –85.44 ppm (Fig. 3E and F). By ^1^H NMR (**6**) was observed to also be a potent psBBOX* inhibitor (IC_50_ = 1.6 μM, Fig. S15, ESI†). Titration of (**6**) with psBBOX*-Zn^(II)^-GBB manifested concentration dependent decrease of the putative psBBOX*-Zn^(II)^-GBB signal at –82.19 ppm, concomitant with increases in the assigned psBBOX*-Zn^(II)^-(**6**) signals at –85.18 and –85.44 ppm. A similar result was obtained with another potent hBBOX inhibitor, AR692B (**7**) (IC_50 hBBOX_ = 0.2 μM),^32^ with concentration dependent formation of signals at –85.19 and –85.43 ppm and attenuation of the psBBOX*-Zn^(II)^ signal at –85.34 ppm being observed (Fig. 3G). The two signals obtained with (**6**) and (**7**) may reflect different binding modes which the ligand can adopt in the same dimer, as observed by crystallography with (**7**) with hBBOX (Fig. 3H).^32^ Note that, at least by ^1^H NMR, (**7**) appears to be a relatively poor inhibitor of psBBOX* (IC_50_ = 245 μM, Fig. S15, ESI†) compared to wt-psBBOX, suggesting psBBOX* is an imperfect model for hBBOX.

The combined NMR studies on ligand binding led to the proposal that a new BBOX inhibitor scaffold could be identified by directly incorporating analogues of identified 2OG and GBB binding motifs, comprising appropriately positioned metal chelating and quaternary ammonium moieties, giving a ‘co-substrate/substrate’ mimic. With this in mind, RL190B (**8**) was synthesised (ESI†); it is a relatively potent psBBOX inhibitor (IC_50_ = 5 μM (fluoride release assay)^31^). Inhibition of psBBOX* by (**8**) was confirmed by ^1^H NMR (IC_50_ = 15 μM Fig. S15, ESI†). Pleasingly, (**8**) was observed to displace both GBB and 2OG from the psBBOX active site using a ^1^H NMR reporter assay (Fig. 4A); ^19^F NMR titrations of (**8**) with psBBOX*-Zn^(II)^ showed significant broadening and almost complete attenuation of the original psBBOX* signal (Fig. 4C). By contrast with other ligand titrations using psBBOX* such significant line broadening was not observed (*e.g.* Fig. S11–S13, ESI†). Broadening of signals in PrOF NMR is often typical of weaker inhibitors; the significant (and unusual amongst studied compounds) signal attenuation observed with (**8**) may be a result of its binding in both 2OG and GBB cavities. This may cause changes of conformational mobility/protein destabilisation, potentially yielding a number of indistinct conformational states.

**Fig. 4:**
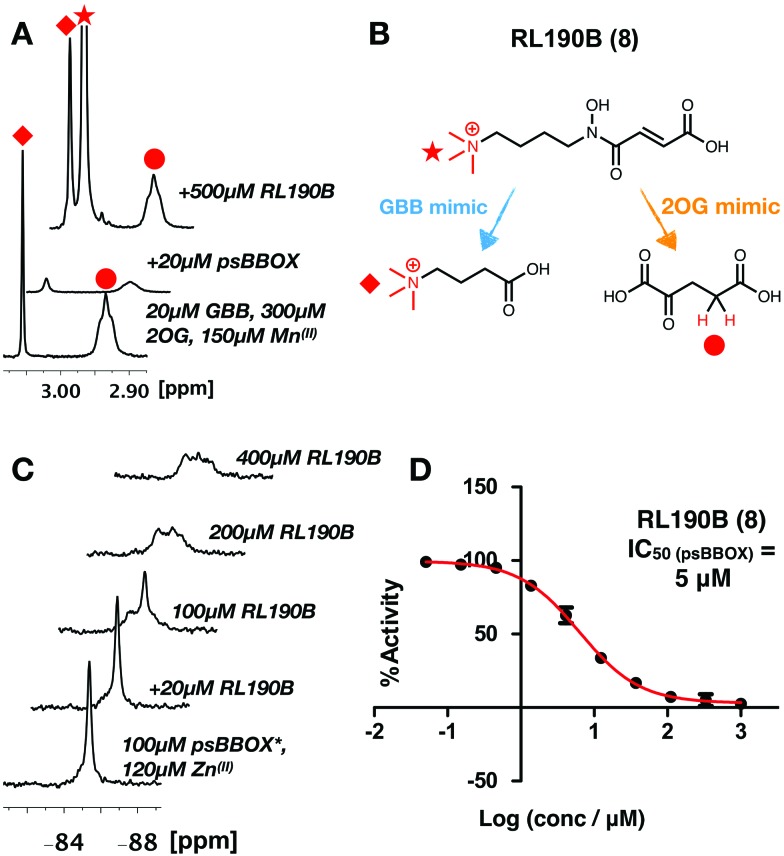
(A) ^1^H reporter assay showing RL190B (**8**) displaces both GBB and 2OG from wild-type psBBOX. (B) RL190B is a 2OG and substrate mimic. (C) RL190B causes attenuation of the psBBOX* ^19^F NMR signal. (D) RL190B inhibition of wt-psBBOX as determined by a fluoride release assay.^31^

The combined results highlight the power of PrOF to reveal insights into cooperative binding, especially when combined with ligand observed NMR. Although such information can be obtained by other methods, including classic kinetics and other biophysical methods (*e.g.* isothermal calorimetry), such methods are often labour intensive and not always applicable. The value of the NMR methods in medicinal chemistry is exemplified by their use in identifying a new type of BBOX inhibitor, suitable for development.

We thank the Biotechnology and Biological Sciences Research Council (BBSRC, BB/E527620/1), Cancer Research UK (C8717/A18245), the Wellcome Trust (091857/7/10/7/099141/Z/12/Z) for funding. We thank the Engineering and Physical Sciences Research Council for a studentship to JK *via* the Centre for Doctoral Training in Synthesis for Biology and Medicine (EP/L015838/1), and a Clarendon Scholarship.

## Conflicts of interest

There are no conflicts to declare.

## Supplementary Material

Supplementary information
