# Supplementary material for: 19F NMR studies on γ-butyrobetaine hydroxylase provide mechanistic insights and suggest a dual inhibition mode
Source: Chem Commun (Camb). 2019 Nov 8;55(98):14717–20. doi: 10.1039/c9cc06466d (PMC6927413; doi:10.1039/c9cc06466d)
Supplement: Supplementary file 1 [file CC-055-C9CC06466D-s001.pdf]

Electronic Supplementary Material (ESI) for Chemical Communications.
This journal is © The Royal Society of Chemistry 2019

†Electronic Supplementary Information (ESI)

^{19}F NMR studies on γ-Butyrobetaine Hydroxylase provide mechanistic insights and enable a new inhibition mode

Robert K Leśniak, Anna M Rydzik,‡ Jos J. A. G. Kamps,‡ Amjad Kahn, Timothy D. W. Claridge,* and Christopher J. Schofield*

The Department of Chemistry, University of Oxford, 12 Mansfield Road, Oxford, OX1 3TA, United Kingdom.

E-mail: christopher.schofield@chem.ox.ac.uk; tim.claridge@chem.ox.ac.uk

Fax: +44 1865 285002, Tel: + 44 1865 275658

‡These authors contributed equally.

Table of Contents

Author Contributions 3
General Experimental Methods 4
Reagents for molecular biology 4
The psBBOX DNA sequence used was: 4
Mutagenesis protocol 5
PCR parameters: 5
Expression studies 5
Cell lysis and protein purification[1] 6
^{19}F labelling of psBBOX-Y201C 7
NMR materials and methods[2] 8
psBBOX Differential Scanning Fluorimetry (DSF) assays 9
Supplementary Figures 10
Figure S0. SDS-PAGE of purified psBBOX Y201C 10
Figure S1. MS analysis of wild-type psBBOX 11
Figure S2. MS analysis of psBBOX-Y201C labelling 11
Figure S3. ^{1}H NMR Turnover assays. 12
Figure S4. psBBOX* Timecourse analysis 12
Figure S5. Kinetic Characterisation of psBBOX-Y201C-BFA (psBBOX*). 13
Figure S6. Differential Scanning Fluorimetry (DSF) assay results. 14
Figure S7. Titrations of Ni(II) and Zn(II) with psBBOX* 15
Figure S8. Titration of 2OG with psBBOX*-Zn(II) 16
Figure S9. ^{1}H binding assay of GBB to psBBOX-Y201C-BFA (psBBOX*). 17
Figure S10. Inhibition and binding of Mildronate (4) to psBBOX-Y201C-BFA (psBBOX*) using ^{1}H NMR. 17
Figure S14. Titration of DMSO with psBBOX*-Zn$^{(II)}$. 21
.... 21
Figure S15. ^{1}H NMR Inhibition assay of psBBOX-Y201C-BFA (psBBOX*) with (6), (7) and RL190B (8).... 22
Synthesis 23
Materials and methods 23
Synthesis of RL190B 24
Tert-Butyl (4-((benzoyloxy)amino)butyl)carbamate (2) 24
Ethyl (E)-4-((benzoyloxy)(4-((tert-butoxycarbonyl)amino)butyl)amino)-4-oxobut-2-enoate (3) 25
Ethyl (*E*)-4-((4-aminobutyl)(benzoyloxy)amino)-4-oxobut-2-enoate (4) 26
(*E*)-4-(*N*-(Benzoyloxy)-4-ethoxy-4-oxobut-2-enamido)-*N,N,N*-trimethylbutan-1-aminium (5) 26
(*E*)-4-(3-Carboxy-N-hydroxyacrylamido)-*N,N,N*-trimethylbutan-1-aminium (RL190B) (6) 27
References 28

Author Contributions

R.L.K. carried out all the synthetic aspects of the work and most of the assay and ^{19}F NMR work; he also carried out protein preparations. A.M.R. suggested and pioneered the ^{19}F labelling approach used, prepared wildtype and variant proteins, carried out thermal shift and MS studies, and guided NMR experimental and inhibitor design. A.K carried out NMR studies involving non-fluorinated protein leading to evidence for cooperativity. J.J.A.G.K. carried out kinetic assays, and inhibitor and binding studies by ^{1}H NMR. T.D.W.C. supervised NMR aspects of the work. All authors analysed data. C.J.S. supervised the project and with J.J.A.G.K and R.L.K. wrote drafts of the manuscript, which was ultimately approved by all authors.

General Experimental Methods

Reagents for molecular biology

Materials and reagents were from New England Biolabs (NEB), Invitrogen, Bio-Rad, Roche, or Simga-Aldrich, unless otherwise stated. Water was initially purified using a Millipore Elix® Reverse Osmosis system, then using a Millipore Milli-Q® Synthesis system, with a 0.22 µm filter. IPTG and ampicillin solutions were sterilized before use with a 0.2 µm filter from Minisart®, Sartorius Stedim Biotech.

The psBBOX DNA sequence used was:

CATATGAATGCGATTGCAGATTATCGTACCTTTCCGCTGATTAGTCCGCTGGCAAGCGCAGCC
AGCTTTGCAAGCGGTGTTAGCGTTACCTGGGCAGATGGTCGTGTTAGCCCGTTTCATAATCTG
TGGCTGCGTGATAATTGTCCGTGTGGTGATTGTGTTTATGAAGTTACCCGTGAACAGGTTTTTC
TGGTTGCAGATGTTCCGGAAGATATTCAGGTTCAGGCAGTTACCATTGGTGATGATGGTCGTC
TGGTTGTTCAGTGGGATGATGGCCATGCAAGCGCATATCATCCGGGTTGGCTGCGTGCACATG
CCTATGATGCACAGAGCCTGGCAGAACGTGAAGCAGCACGTCCGCATAAACATCGTTGGATG
CAGGGTCTGAGCCTGCCGGTTTATGATCATGGTGCAGTTATGCAGGATGATGATACCCTGCTG
GAATGGCTGCTGGCAGTTCGTGATGTTGGTCTGACCCAGCTGCATGGTGTTCCGACCGAACCG
GGTGCACTGATTCCGCTGGCCAAACGTATTAGCTTTATTCGCGAAAGCAATTTTGGCGTGCTG
TTTGATGTTCGTAGCAAAGCAGATGCAGATAGCAATGCA**TAT**ACCGCATTTAATCTGCCGCTG
CATACCGATCTGCCGACACGTGAACTGCAGCCTGGTCTGCAATTTCTGCATTGTCTGGTTAAT
GATGCAACCGGTGGTAATAGCACCTTTGTTGATGGTTTTGCAATTGCCGAAGCACTGCGTATT
GAAGCACCGGCAGCATATCGTCTGCTGTGTGAAACACCGGTTGAATTTCGTAATAAAGATCG
CCATAGCGATTATCGTTGTACCGCACCGGTTATTGCACTGGATAGCAGCGGTGAAGTTCGTGA
AATTCGTCTGGCAAATTTTCTGCGTGCTCCGTTTCAGATGGATGCACAGCGTATGCCGGATTA
TTATCTGGCCTATCGTCGTTTTATTCAGATGACGCGTGAACCGCGTTTTTGTTTTACCCGTCGT
CTGGAAGCAGGTCAGCTGTGGTGTTTTGATAATCGTCGTGTTCTGCATGCACGTGATGCCTTT
GATCCGGCAAGTGGTGATCGTCATTTTCAGGGTTGTTATGTTGATCGTGATGAACTGCTGAGC
CGTATTCTGGTTCTGCAGC GTTAAGGAT

Red letters highlight site of mutagenesis.

Mutagenesis primers were designed using an online tool:

https://www.genomics.agilent.com/CollectionSubpage.aspx?PageType=Tool&SubPageType=ToolQCPD&PageID=1 5.

Primers for the psBBOX Y201C variant were as follows:

Forward primer: 5'-caaagcagatgcagatagcaatgcatgtaccgcatttaatc-3' ;

Reverse primer: 5'-gattaaatgcggtacatgcattgctatctgcatctgctttg-3'.

Mutagenesis protocol

The PCR mixture for mutagenesis was: 1 µL psBBOX template (psBBOX in pCOLD 10 ng/µL); 2 µL primer 1 (10 µM); 2 µL primer 2 (10 µM); 5 µL Pfu buffer (10×); 5 µL dNTP mix (2 mM); 1 µL Pfu Turbo polymerase; 34 µL H_2O.

PCR parameters:

Supplementary Table 1: Parameters for PCR.

Step	Time	Temperature (°C)	
Initial hold	30 sec	95	
Denaturing	30 sec	95	16 Cycles
Annealing	60 sec	55	
Extension	6 min	68	
Final Extension	10 min	68	

After PCR reactions, samples were digested using DpnI (1.5 µL, 4h, 37°C). The resultant mixtures were transformed into XL10 GOLD competent cells (New England Biolabs, transformation according to the manufacturer's protocol) and plated on agar plates containing ampicillin and incubated overnight at 37°C. DNA from colonies was isolated using a miniprep kit (GeneJet) and sequenced. DNA was then transformed into BL21 (DE3) competent cells. Large scale growths and purifications were carried out according to the protocol used for wild-type psBBOX.[1]

Expression studies

DNA encoding for psBBOX-Y201C with an *N*-terminal hexa his-tag in a pCOLDI construct was transformed into BL21 (DE3) competent cells, which were frozen as a glycerol stock in liquid nitrogen and stored at -80°C. Glycerol stocks were made up using a 3:1 ratio of glycerol to cell culture. An overnight starter culture was prepared and large-scale recombinant protein production and purification was carried out according to the protocol for wild-type psBBOX.[1] 12 flasks containing 2TY media (600 mL) were supplemented with ampicillin (50µg/mL) before 6mL of the overnight starter culture was added. The flasks were then incubated at 37°C with shaking (180 rpm) until the OD_{600} reached 0.6. The temperature was then reduced to 15°C

and shaking stopped. The flasks were kept at this temperature for 30 mins then IPTG (ispropyl β–galactopyranoside) was added (0.2 mM final concentration). Flasks were then incubated overnight (15°C with shaking). The bacterial cultures were then dispensed into centrifuge tubes and pelleted via centrifugation (10 min, 7000 rpm). The resulting cell pellet was transferred into plastic sealable bags, weighed, and frozen at -80°C.

2TY (2X Tryptone/Yeast) medium used for bacterial growth was made up of: 16g Bacto typtone, 10g Yeast extracts, 5g NaCl. Media was autoclaved at 121°C for 20 minutes before use. *E. coli* strain used: *BL21 (DE3)*: F$^-$ *ompT hsdS*$_B$ ($r_B^- m_B^-$) *gal dcm* (DE3).

Cell lysis and protein purification[1]

A frozen cell pellet was placed in an ice cooled glass beaker equipped with a stirrer bar. The His column binding buffer was added to the cell pellet (5 × weight of frozen cells), followed by 1 mg of DNAse I and a protease inhibitor tablet (Roche). Stirring was continued until the cell pellets had thawed. The resultant suspension was subjected to sonication (10 seconds on 10 seconds off pulse sequences for a total of 10 minutes); using a Vibra Cell VCX 500 sonicator equipped with a 13 mm probe. The resultant suspension was centrifuged (14,000 rpm, 20 mins, 4 °C); the supernatant was then collected, and filtered using a 0.4 µm Omnipore™ filter (Millipore U.K.).

The filtered protein was then purified using an Amersham Pharmacia Biotech Äkta FPLC System (P920 pump system, IPC900 UV detector, Frac900 fraction collector and Unicorn Sotware) and 5 mL nickel affinity column (His-Trap®, Novagen).[1] The His-Trap® column was equilibrated with 10 column volumes (CV) of binding buffer. The filtered cell lysate was loaded onto the column using an FPLC pump, which was washed with 5 CVs of binding buffer. The column was then washed with 5-10 CV (until the UV absorbance reached base line levels) of wash buffer. The bound protein was eluted with 5CV of elution buffer and fraction collection. UV absorbing fractions were analysed by SDS-PAGE. Fractions containing protein were treated with EDTA (final concentration 1 mM) and left overnight at 4°C. The fractions were then concentrated using an Amicon® Ultra-4 Ultracel® PL filtration device (Millipore) with 30 kDa molecular weight limit membranes and an Allegra™ 21R centrifuge (Beckman Coulter, S4180 rotor, 3500 rpm, 4°C). The resultant protein solutions were then buffer exchanged into

Tris buffer using a PD10 desalting column (GE Healthcare) following the manufacturers protocol. On average 85 mg of protein per L of culture media was obtained from 12 flasks of media.

Buffers used during His-column purification:
Binding buffer: 50 mM HEPES, pH 7.6, 0.5 M NaCl, 5 mM imidazole;
Wash buffer: 50 mM HEPES, pH 7.6, 0.5 M NaCl, 30 mM imidazole;
Elution buffer: 50 mM HEPES, pH 7.6, 0.5 M NaCl, 500 mM imidazole;
Strip buffer: 50 mM HEPES, pH 7.6, 0.5 M NaCl, 100 mM imidazole;
Tris buffer: 50 mM Tris, pH 7.5, 0.2 M NaCl.

^{19}F labelling of psBBOX-Y201C

The reported procedure for BFA labelling was used.[2]
A psBBOX-Y201C solution at 5°C was buffer exchanged into PBS buffer. A fresh solution of BFA was made up in PBS buffer (100 mM). The BFA PBS solution was added to the psBBOX-Y201C solution to give a 12.5 : 1 ratio of BFA : Y201C. The resulting mixture was left at room temperature for 10 minutes then buffer exchanged into Tris buffer using a PD-10 desalting column. The resulting protein in tris buffer was concentrated, aliquoted and frozen and stored at -80°C.

The following protein labelling MS conditions were used:

A Waters LCT instrument fitted with a TOF analyser, ESI ionisation mode, and an HP1100 chromatography system (Agilent), with a ProSwift RP-4H analytical column (1 × 50 mm, Thermo) was used for analysing the ^{19}F labelled protein. The following solvents were used: solvent A – water 0.1% (v/v) formic acid, solvent B – acetonitrile 0.1% (v/v) formic acid.

Supplementary Table 2 : Solvent gradient for MS analysis.

Time (min)	% A	Flow rate [ml/min]
0.00	95	0.4
0.50	95	0.4
4.50	5	0.4
6.50	5	0.4
7.50	95	0.4
10.00	95	0.4

Protein spectra were calibrated using myoglobin (mass accuracy <5 ppm). Tuning file parameters: sample cone voltage; 25 V, extraction cone voltage; 5 V, RF lens 255, desolvation temperature: 300 °C, source temperature: 100 °C; MS method: 0-10 min, mass range 300-1800, ES+, Tof MS, MS scan mode; Pressure limit: 2500 psi.

NMR materials and methods[2]

NMR measurements used a Bruker AVIII 600 spectrometer equipped with a BB-$^{19}F/^{1}H$ Prodigy N_2 cryoprobe operating at 298 K using 5 mm diameter NMR tubes (Norell). Lorentzian Line broadening of 20 Hz was applied using MestReNova version 11.0. Spectra were referenced to the internal 1,1,1-trifluoroacetic acid standard (TFA, 10 or 50 µM, -76.55 ppm).

A typical NMR sample had a final volume of 500 µL in a 5 mm diameter NMR tube. Samples contained: 100 µM psBBOX* in Tris buffer (50 mM, pH 7.5) and 200 mM NaCl in 90% H_2O and 10% D_2O (50 µL).

psBBOX Differential Scanning Fluorimetry (DSF) assays

See also Supplementary Figure S5.

10 µL of 250 µM stock (5 ×) of metal in 50 mM HEPES pH 7.5 was added to well (final concentration 50 µM). Controls contained 10 µL of buffer instead of metal solution. 2 mL of master mix was prepared by mixing 1.25 mL stock of sypro orange (made by diluting 1 µL of sypro orange in 2.5 mL of 50 mM HEPES pH 7.5), 11 µL of 460 µM psBBOX (final conc 2 µM) and 739 µl of 50 mM HEPES pH 7.5. 40 µL of mix was added to wells, to get final volume of 50 µL. Metal solutions were made freshly. Experiments was carried out in triplicate.

DSF was performed using a MiniOpticon Real-Time PCR Detection System (Bio-Rad), monitoring protein unfolding using SYPRO orange (Invitrogen) according to reported method.[3] 6-Carboxyfluorescein (FAM™) (492 nm) and 6-carboxyl-X-Rhodamine (ROX™) (610 nm) filters were used for excitation and the emission, respectively. Reaction mixtures contained: 2 µM protein, 50 µM metal and 1 × SYPRO orange (final volume 50 µL). Reagents were prepared in 50 mM HEPES buffer pH 7.5. Iron solution was prepared shortly before experiment. Fluorescence readings were taken every 1 °C in the range 25−95 °C, with the temperature increased linearly by 1 °C per minute.

The inflection point, representing T_m, was calculated by fitting the Boltzmann equation to the sigmoidal curves obtained; data were processed using GraphPad Prism 5.0. The T_m shift caused by the addition of small molecules/fragments was determined by subtraction of the “reference” T_m (protein without metal) from the T_m obtained in the presence of the metal. Conditions were tested in triplicate, with standard deviations typically <1 °C.

Supplementary Figures

Figure S0. SDS-PAGE of purified psBBOX Y201C. Molecular weight markers (Thermo Scientific PageRuler Prestained Protein Ladder 10-170 kDa).

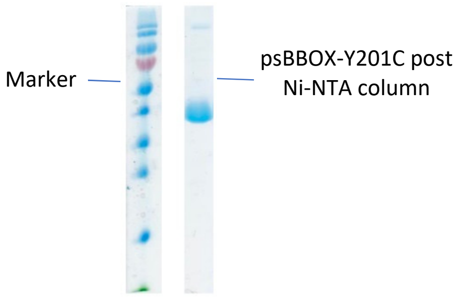

Figure S1. MS analysis of wild-type psBBOX. MS spectrum of wild-type psBBOX (mass = 45345 Da) (A) and MS spectrum of wt-psBBOX after incubation with 100 equivalents of BFA (obtained mass = 45345 Da) (B). Note the lack of a mass shift.

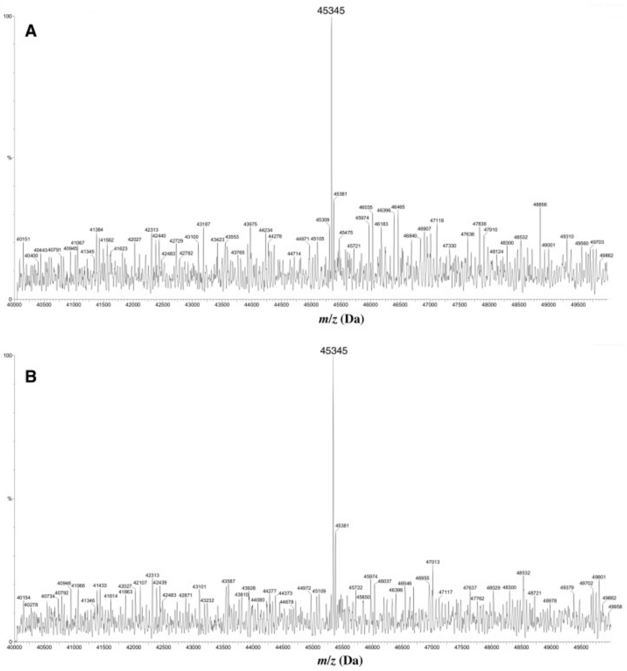

Figure S2. MS analysis of psBBOX-Y201C labelling. MS spectra of psBBOX Y201C labeling. (A) psBBOX Y201C (mass = 45285 Da). (B) psBBOX Y201C, after incubation with 100 equivalents of BFA (obtained mass = 45413 Da). Note the mass shift of 128 Da corresponding to labelling with BFA.

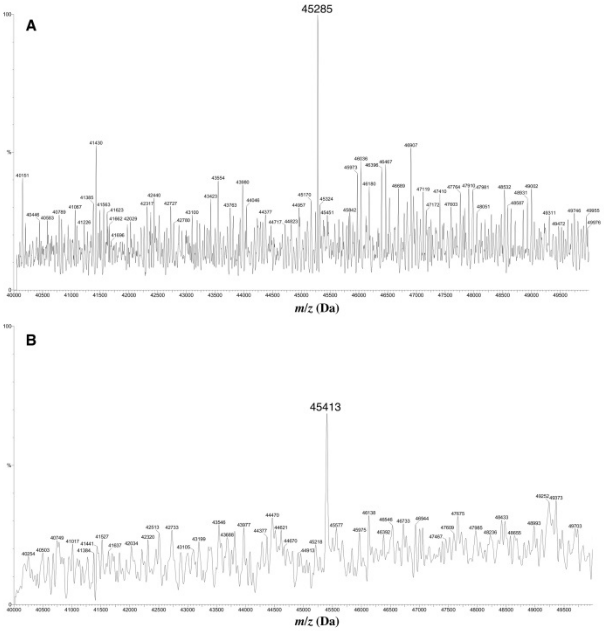

Figure S3. ^{1}H NMR Turnover assays. ^{1}H NMR timecourse experiment. The first spectrum (bottom, red) was acquired without the presence of $Fe^{(II)}$ and psBBOX*. The other spectra show GBB hydroxylation and concomitant succinate formation at 0.03 and 0.02 μM·s^{-1}, respectively. Conditions: 1 μM psBBOX*, 500 μM $Fe^{(II)}$ ([$(NH_4)_2Fe(SO_4)_2.6H_2O$] in 20 mM HCl in D_2O), 500 μM GBB (Cl^- salt) , 250 μM 2OG, 250 μM L-ascorbate (di-sodium salt).

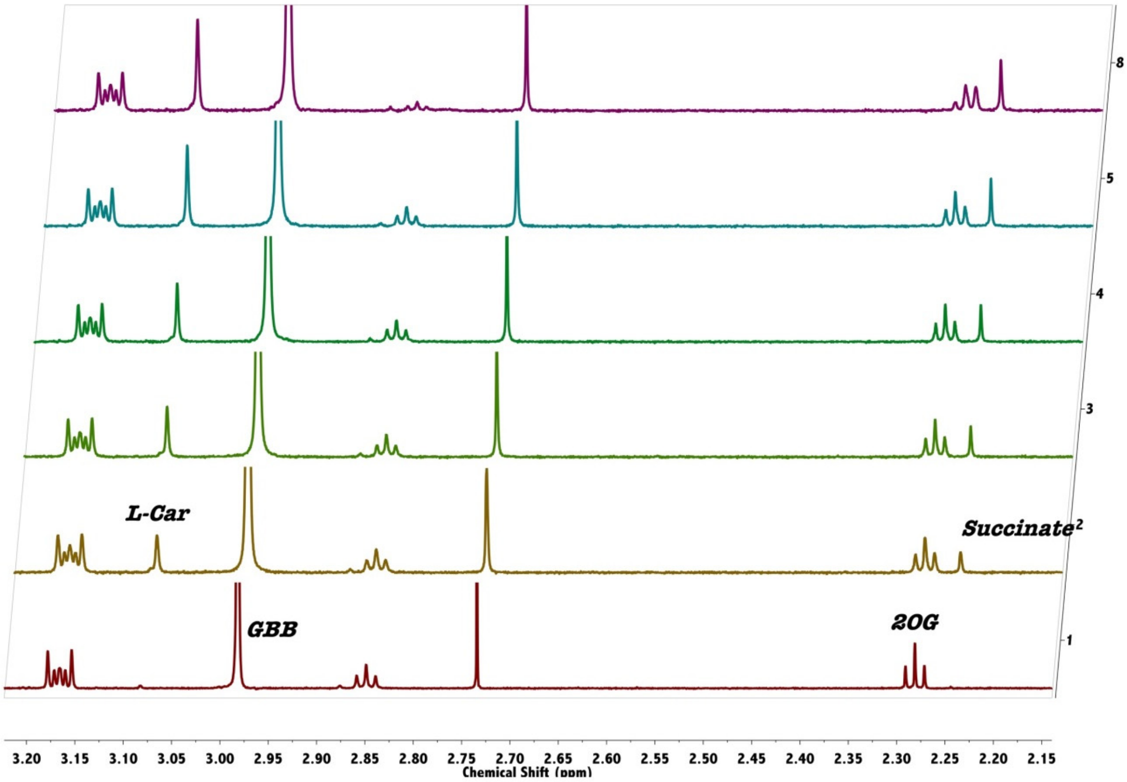

Figure S4. psBBOX* Timecourse analysis of psBBOX-Y201C-BFA (psBBOX*) catalysed hydroxylation of GBB as observed by carnitine and succinate formation. Conditions: 1 μM psBBOX*, 500 μM $Fe^{(II)}$ ([$(NH_4)_2Fe(SO_4)_2.6H_2O$] in 20 mM HCl in D_2O), 500 μM GBB (Cl^- salt), 250 μM 2OG, 250 μM L-ascorbate (di-sodium salt).

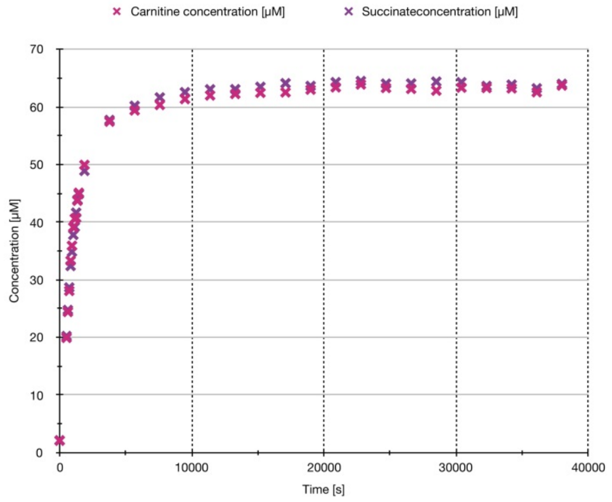

Figure S5. Kinetic Characterisation of psBBOX-Y201C-BFA (psBBOX*).

(**A**) ***wt-psBBOX conditions***: 0.25 µM psBBOX*, 40 µM $Fe^{(II)}$ ([$(NH_4)_2Fe(SO_4)_2.6H_2O$] in 20 mM HCl in D_2O), 2.5 mM 2OG, 500 µM L-ascorbate (di-sodium salt).

(**B**) ***psBBOX* conditions***: 1.86 µM psBBOX*, 40 µM $Fe^{(II)}$ ([$(NH_4)_2Fe(SO_4)_2.6H_2O$] in 20 mM HCl in D_2O), 2.5 mM 2OG, 500 µM L-ascorbate (di-sodium salt).

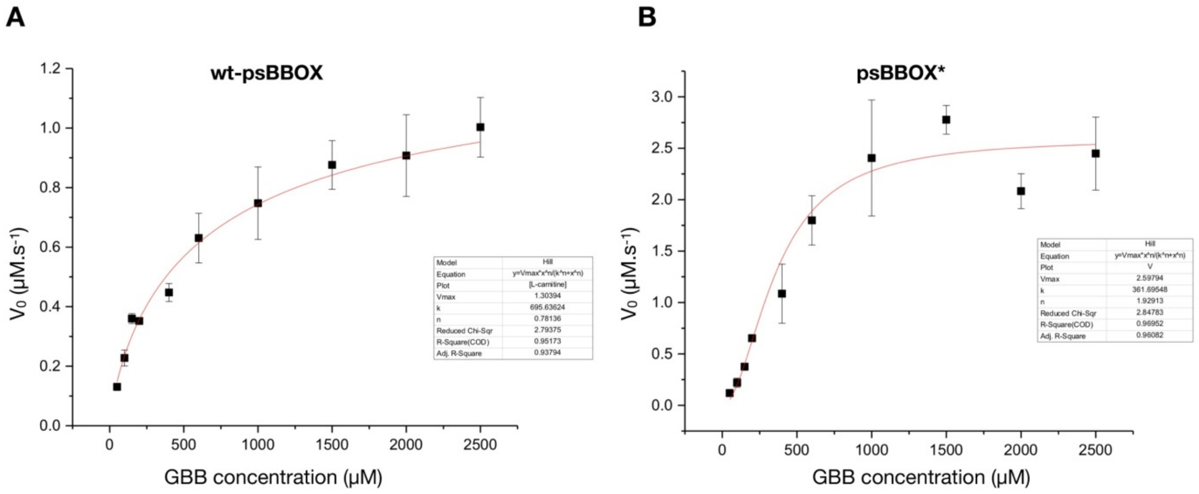

Enzyme	V_{max} (µM·s^{-1})	k_{cat} (s^{-1})	K_m (µM)
wt-psBBOX	1.30 ± 0.41	5.2	700 ± 55
psBBOX*	2.60 ± 0.23	1.4	360 ± 60

Figure S6. Differential Scanning Fluorimetry (DSF) assay results.

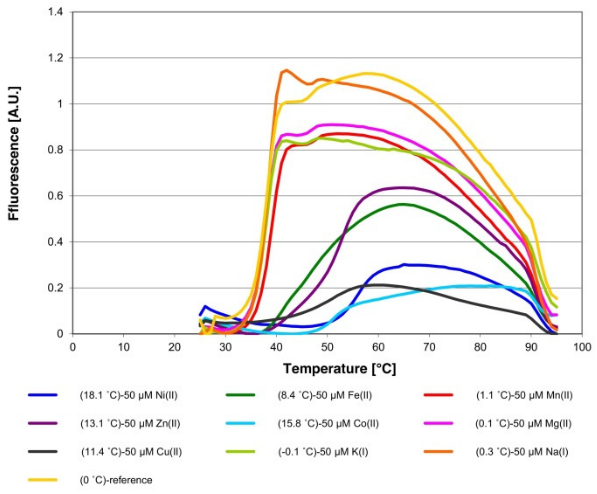

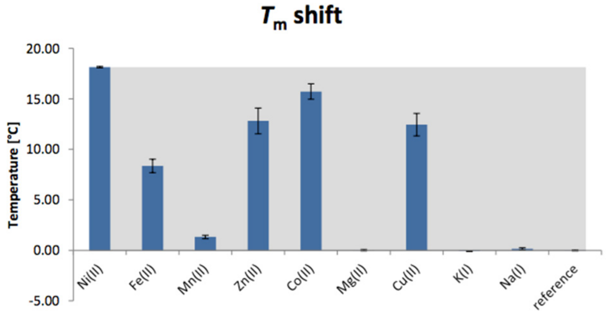

Metal	T_m Shift	T_m	Comment
Ni(II)	18.1±0.1	12.3±0.2	Sulphate salt
Fe(II)	8.4±0.7	5.8±0.4	Ammonium ferrous sulphate
Mn(II)	1.3±0.2	17.7±1.4	Sulphate salt
Zn(II)	12.8±1.3	6.6±0.7	Sulphate salt
Co(II)	15.7±0.8	6.6±2.2	Chloride salt
Mg(II)	0.0±0.1	20.1±0.2	Sulphate salt
Cu(II)	12.4±1.1	6.3±0.5	Sulphate salt
K(I)	0.0±0.1	21.2±0.6	Chloride salt
Na(I)	0.2±0.1	20.5±0.4	Chloride salt
No Metal	0.0±0.1	19.3±1.3	Just Buffer

Figure S7. Titrations of Ni(II) and Zn(II) with psBBOX*. ^{19}F NMR spectra showing effects of (**A**) Ni$^{(II)}$ and (**B**) Zn$^{(II)}$ on psBBOX*. A) Titration of Ni$^{(II)}$ induces shift of the original signal to -85.28 ppm. B) Addition of up to 1.2 equivalents (120 μM) of $ZnCl_2$ induces line broadening.

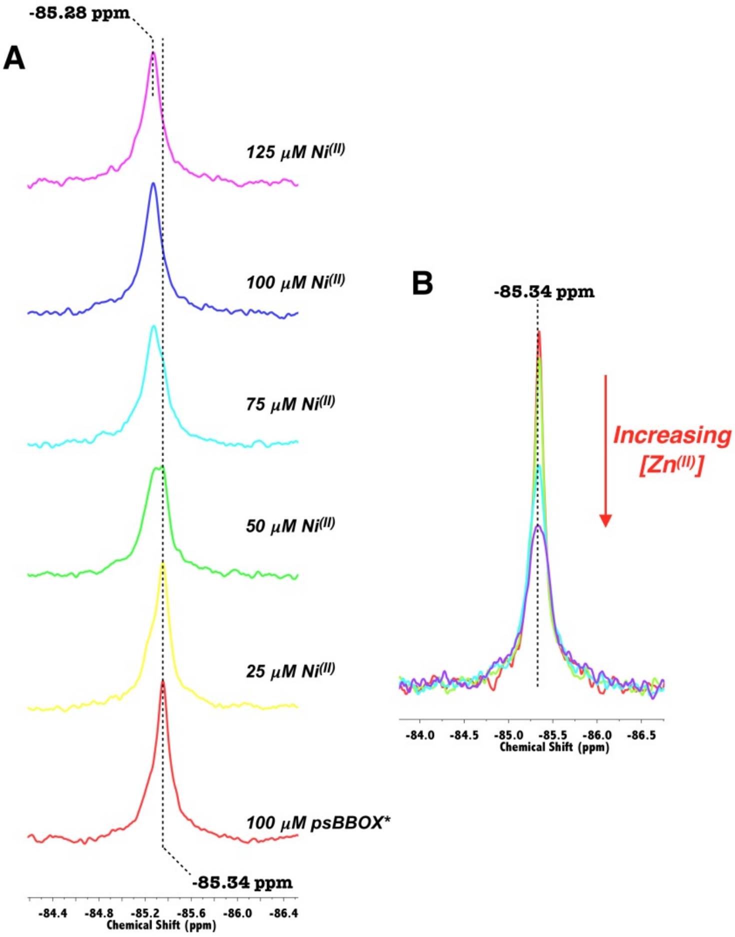

Figure S8. Titration of 2OG with psBBOX*-Zn(II). ^{19}F NMR spectra of titrations of Zn(II) and 2OG with psBBOX*. A) Increasing 2OG concentrations induce signal broadening. B) An overlaid view of the spectra in (A).

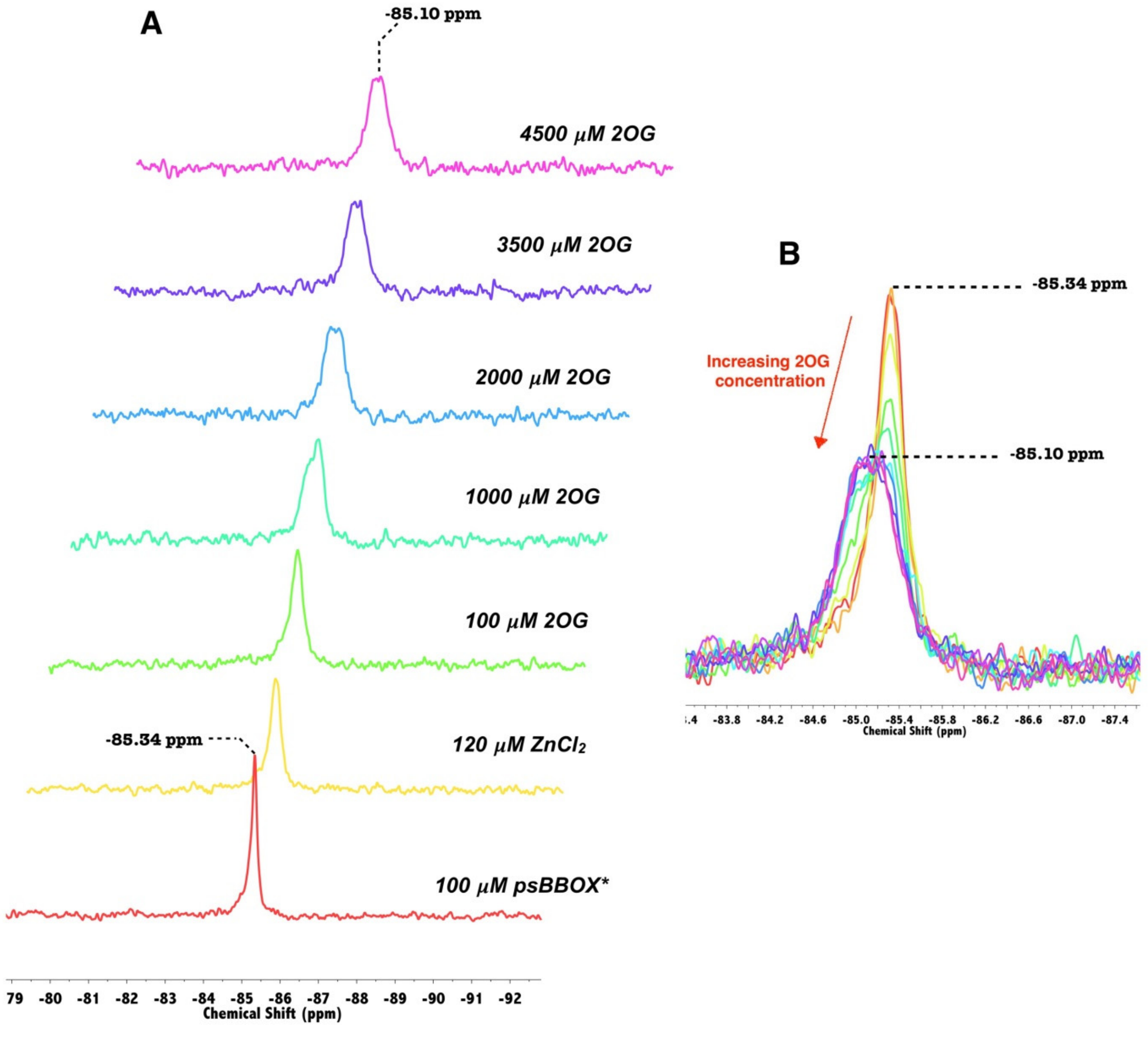

Figure S9. [1]H binding assay of GBB to psBBOX-Y201C-BFA (psBBOX*).
Conditions 25 µM GBB, 150 µM $MnSO_4$, 300 µM 2OG, in phosphate buffer (50 mM, pH 7.1, pD 7.5, in D_2O). K_D = 8.3 ± 0.5 µM

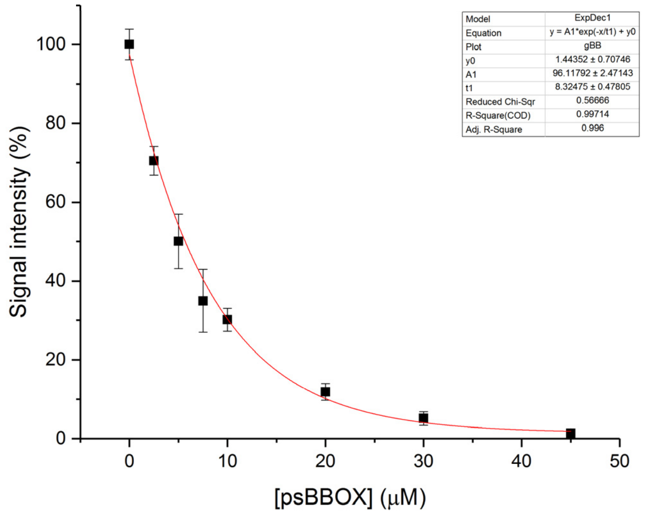

Figure S10. Inhibition and binding of Mildronate (4) to psBBOX-Y201C-BFA (psBBOX*) using ^{1}H NMR.

(**A**) *Inhibition determination conditions*: 2.5 µM psBBOX*, 100 µM $Fe^{(II)}$ ([$(NH_4)_2Fe(SO_4)_2 \cdot 6H_2O$] in 20 mM HCl in D_2O), 200 µM GBB (Cl^- salt), 600 µM 2OG, in Tris-d_{11} (pH 7.5 in H_2O).

(**B**) *Binding affinity determination conditions*: 25 µM Mildronate, 150 µM $MnSO_4$, 300 µM 2OG, in phosphate buffer (50 mM, pH 7.1, pD 7.5, in D_2O). K_D = 12.3 ± 1 µM.

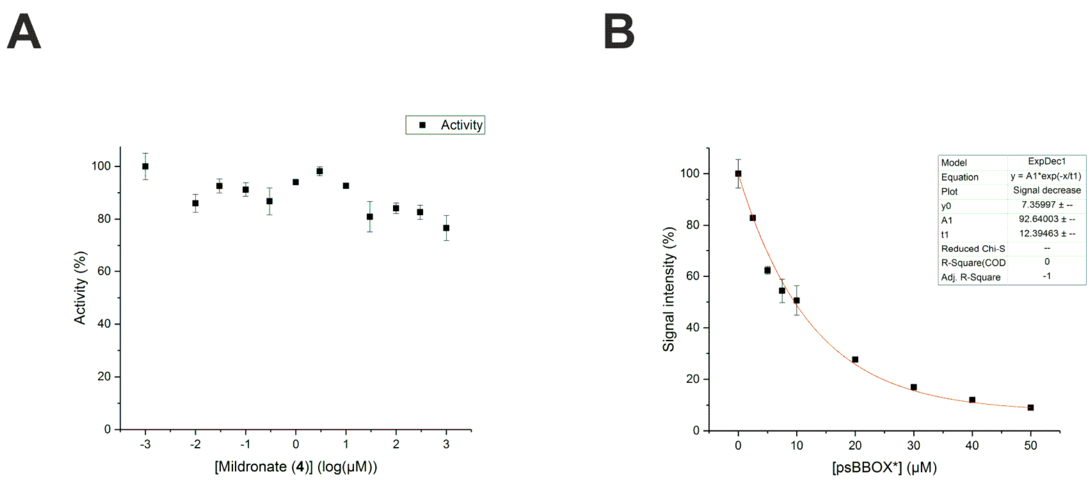

Figure S11. ^{19}F NMR analysis of isoquinoline inhibitors shows sensitivity to differing ligand structures within a series. Monitoring binding of FG2216 analogues to psBBOX* by ^{19}F NMR. Peak shapes obtained from individual titrations of isoquinoline-based inhibitors to psBBOX*-Zn$^{(II)}$ complex are displayed. (Only final 1 mM inhibitor results are displayed; apo-psBBOX* and psBBOX*-Zn$^{(II)}$ spectra are shown for reference.

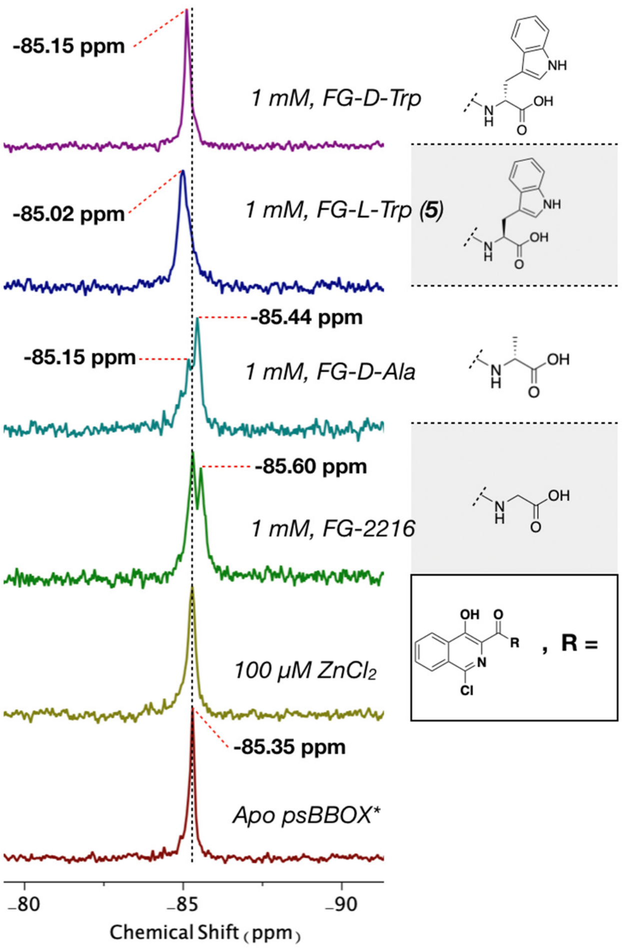

Figure S12 Use of ^{19}F NMR to analyse binding of reported GBB substrate and product analogues to psBBOX*-Zn(II). Resulting signals obtained from two separate titrations of L-carnitine and D-carnitine to psBBOX*-Zn$^{(II)}$ followed by 2OG addition (**A**). Although L-carnitine (but not D-carnitine) was previously determined not to be a substrate for wild-type psBBOX within the assay detection limits,[1] ^{19}F NMR experiments with psBBOX* and L- or D-carnitine, show that 2OG may bind to these complexes. Synthetic analogue P-GBB and GBBNF, are substrates for psBBOX, resulting in the formation of the phospha analogue and fluorinated analogue of L-carnitine respectfully. Their binding in presence of 2OG is observed (**B** and **C**).

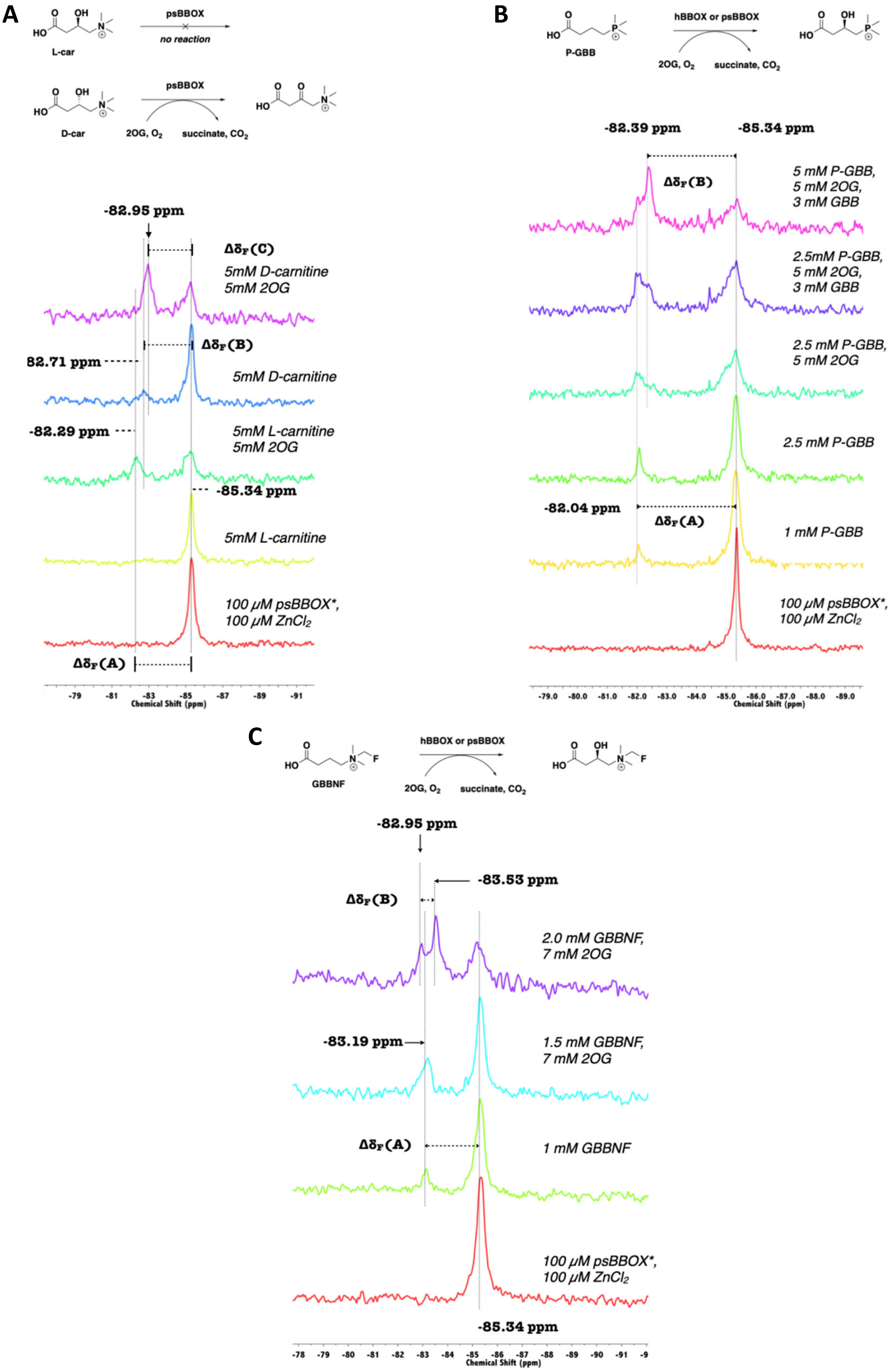

Figure S13 ^{19}F NMR reveals differences in structure by structurally related compounds: 2OG versus N-oxalylglycine (NOG) (A) and AR758 (B) and GBB.

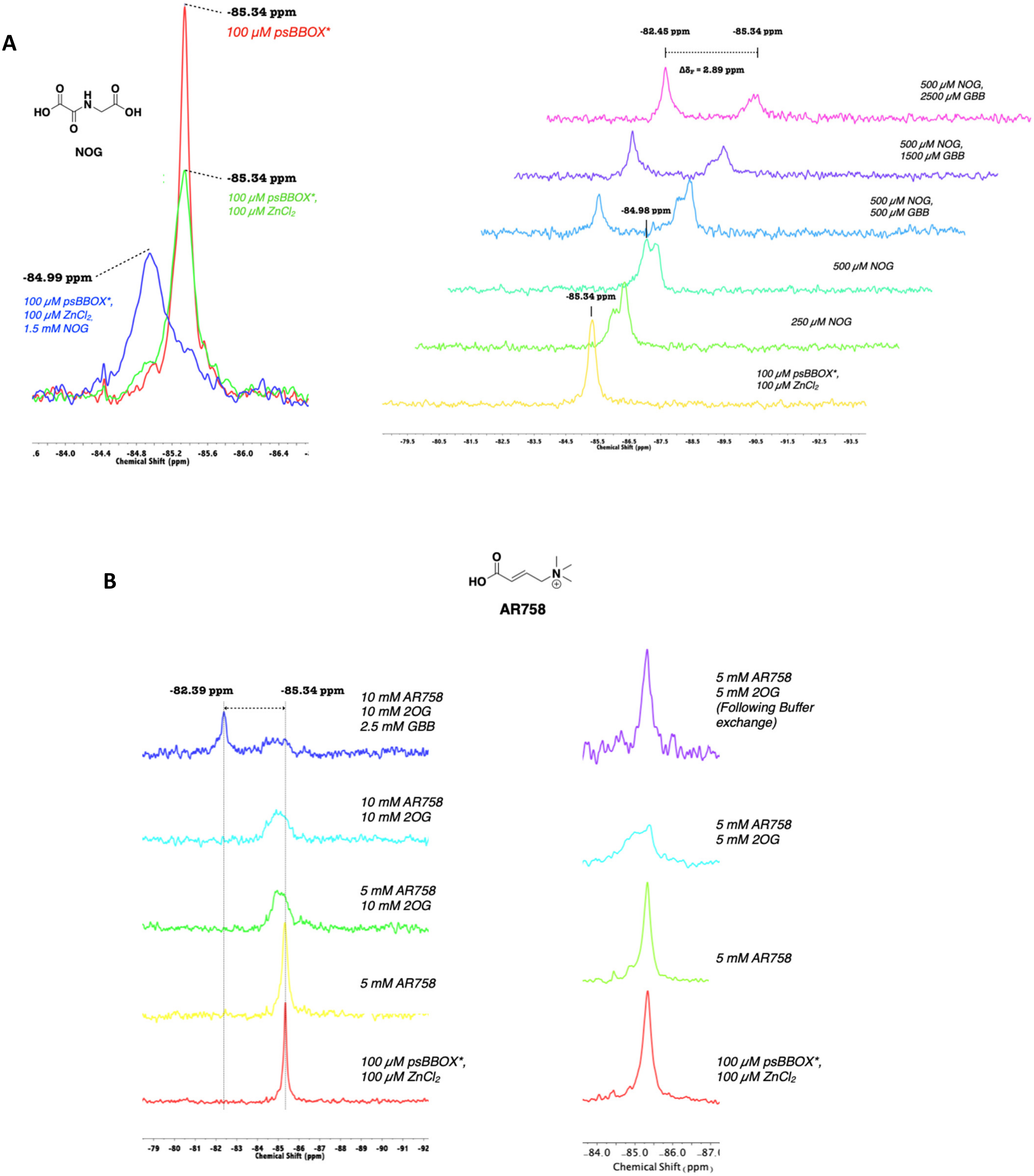

Figure S14. Titration of DMSO with psBBOX*-Zn$^{(II)}$. Graphical representation of the effect of increasing DMSO concentrations on the ^{19}F signal of trifluoroacetic acid and psBBOX*. DMSO appears to have a similar effect on the psBBOX* CF_3 signal as it does on trifluoroacetic acid.

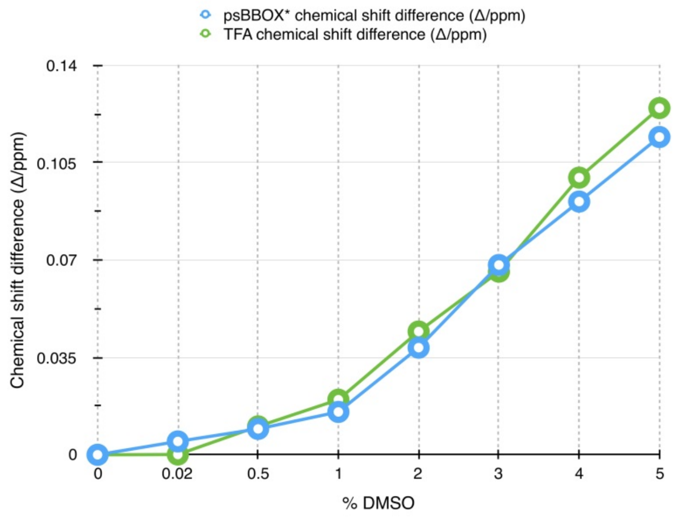

Figure S15. ^{1}H NMR Inhibition assays of psBBOX-Y201C-BFA (psBBOX*) with (6), (7) and RL190B (8).

Assay conditions: 2.5 µM psBBOX*, 200 µM GBB, 10 nM – 1 mM Inhibitor, 100 µM $Fe^{(II)}$ ($[(NH_4)_2Fe(SO_4)_2.6H_2O]$ in 20mM HCl in D_2O), 600 µM 2OG, 500 µM L-ascorbate (di-sodium salt), in Tris-d_{11} (pH 7.5 in H_2O). A) Dose response curve for (**6**), B) dose response curve for (**7**), C) dose response curve for (**8**).

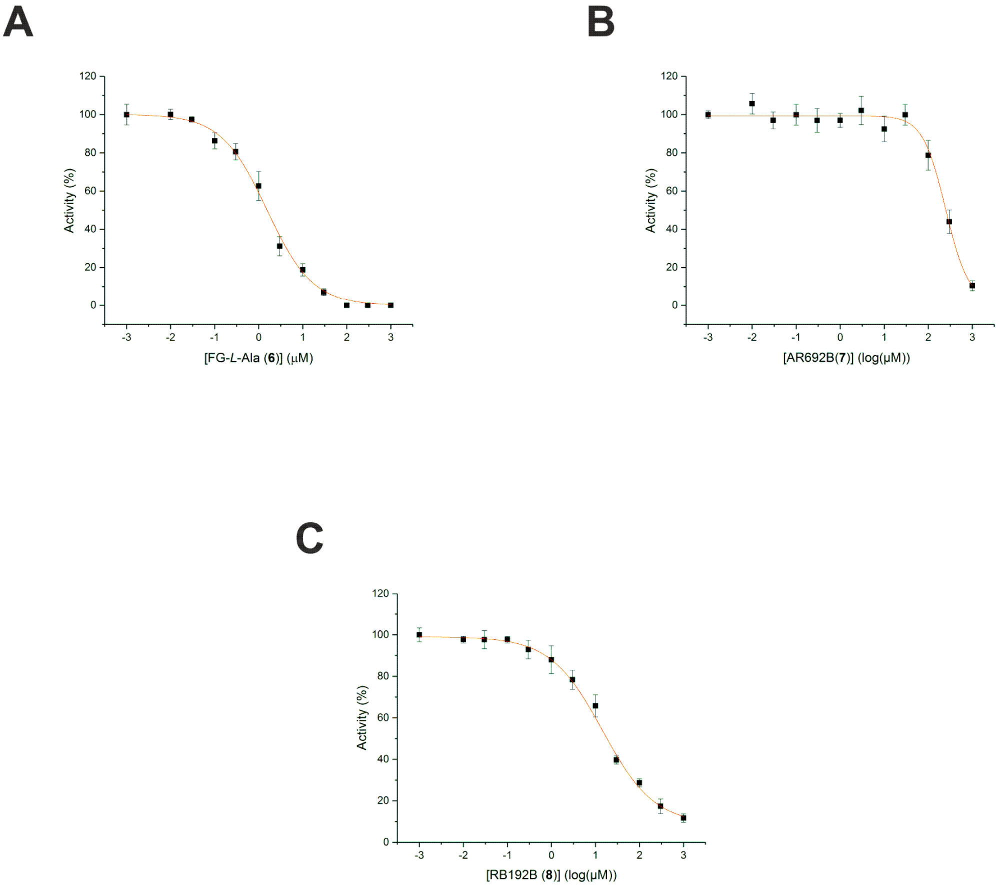

Synthesis

Materials and methods

All chemicals, reagents, and solvents were from Sigma-Aldrich (Dorset, UK) and used without purification. HPLC grade solvents were used for reactions, chromatography, and work-ups. Aqueous solutions were made using de-ionized water. Thin layer chromatography (TLC) was carried out using Merck (Darmstadt, Germany) silica gel 60 F254 TLC plates. TLC visualisation was carried out under UV light and stained with one of three stains: ninhydrin, potassium permanganate, or anisaldehyde. Chromatographic purifications were carried out using a Biotage® (Uppsala, Sweden) Isolera One or Biotage® SP4 purification systems, using Biotage® pre-packed SNAP columns. Reactions were monitored using an Agilent (Cheshire, UK) 1200 series, 6120 quadrupole LC-MS system using a Merck Chromolith® Performance RP-18 HPLC column. Deuterated solvents were from Sigma-Aldrich, and ^{1}H NMR spectra were obtained using a Bruker AVANCE AVIII HD 400 nanobay (400 MHz), Bruker AV500 (500 MHz) with a ^{13}C cryoprobe, and/or Bruker AVIII 700 (700 MHz) with an inverse TCI cryoprobe. All signals are described in δ ppm with multiplets being denoted as singlet, doublet, triplet, quartet, and multiplet using the abbreviations s, d, t, q, and m, respectively. Chemical shifts in presented NMR spectra were referenced using residual solvent peaks with coupling constants, *J*, reported in hertz (Hz) to an accuracy of 0.5 Hz. For high-resolution mass spectrometry (HR-MS), a Bruker MicroTOF instrument with an ESI source and Time of Flight (TOF) analyser was used. MS data are represented as a ratio of mass to charge (m/z) in Daltons. A Bruker Tensor 27 instrument was used to obtain Fourier transform infrared spectra (FT-IR).

Synthesis of RL190B

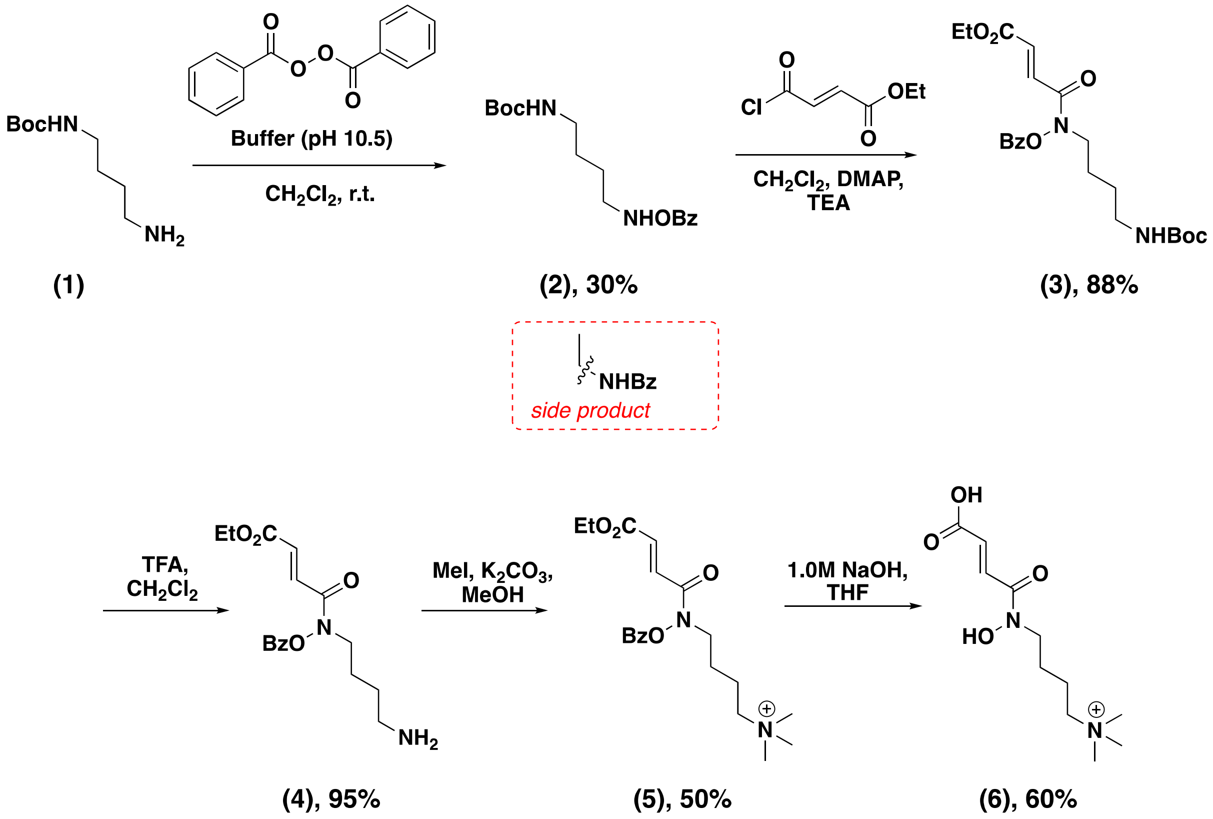

Tert-Butyl (4-((benzoyloxy)amino)butyl)carbamate (2)

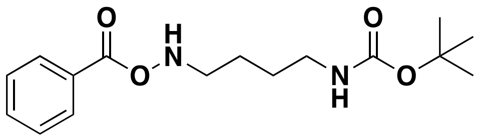

To a buffered (pH 10.5 $NaHCO_3$/NaOH) solution (10 mL) of *N*-tert-butoxycarbonyl-1,4-diaminobutane (**1**) (0.94 g, 5.0 mmol) was added a solution of benzoyl peroxide (1.45 g, 6.0 mmol) in CH_2Cl_2 (20 mL). The mixture was stirred for 2.5 hours at room temperature with monitoring by TLC (5:1, cyclohexane/EtOAc). The mixture was then separated and the aqueous layer extracted with CH_2Cl_2 (3 × 20 mL); the organic extracts were washed with a 10% (aq) $Na_2S_2O_3$ solution, dried over Na_2SO_4, filtered and concentrated to dryness in *vacuo*. The resulting crude oil was purified by chromatography (5:1, cyclohexane/EtOAc), to give a colourless oil (630 mg, 2.04 mmol, 40%). **R_f** 0.1 (5:1, cyclohexane/EtOAc). **^{1}H-NMR** ($CDCl_3$, 400 MHz) δ = **^{1}H NMR** ($CDCl_3$, 400 MHz) δ = 1.43 (9H, s, -^{t}Bu), 1.56 – 1.71 (4H, m, $BocNHCH_2CH_2CH_2CH_2N$-), 3.15 (4H, t, J = 6.5 Hz, $BocNHCH_2CH_2CH_2CH_2N$-), 4.62 (1H, br. t, J =

6.0 Hz, BocN*H*-), 7.43 – 7.48 (2H, m, *Ar*), 7.56 – 7.61 (1H, m, *Ar*), 8.01 (2H, dd, *J* = 8.5, 1.5 Hz, *Ar*) ppm. **^{13}C NMR** ($CDCl_3$, 101 MHz) δ = 24.6, 27.0, 27.7, 28.5, 40.4, 79.3, 128.4, 128.7, 129.5, 133.5, 156.1, 167.0 ppm. **FT-IR** ν_{max} (film) 3336, 2979, 2932, 1689, 1573, 1391, 1270, 1251, 1169, 709 cm^{-1}. **HRMS** (ESI-TOF) calcd for $C_{16}H_{25}N_2O_4$ $[M+H]^+$: 309.1809, found: 309.1811.

Ethyl (E)-4-((benzoyloxy)(4-((tert-butoxycarbonyl)amino)butyl)amino)-4-oxobut-2-enoate (3)

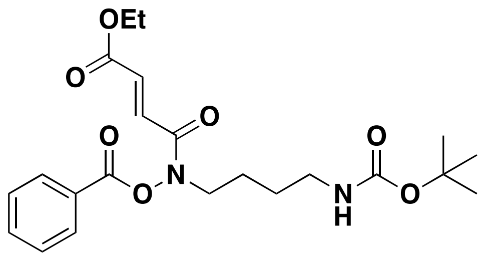

A solution of (**2**) (630 mg, 2.04 mmol) in CH_2Cl_2 (21 mL) was cooled using an ice bath. Triethylamine (0.6 mL, 4.08 mmol), DMAP (2 mg, 0.016 mmol) and ethylfumaroyl chloride (430 mg, 2.65 mmol) were then added sequentially. The mixture was then allowed to warm to room temperature and after 2hrs the starting material was observed to be consumed by TLC (1:1, cyclohexane/EtOAc). Following addition of sat. (aq) NH_4Cl, the aqueous layer was extracted with EtOAc (3 × 20 mL). The organic phase was dried over anhydrous Na_2SO_4, filtered, concentrated and purified by flash column chromatography (10:1, cyclohexane: EtOAc), to give a yellow oil (777 mg, 1.79 mmol, 88%). **R_f** 0.55 (100% EtOAc). **^{1}H NMR** ($CDCl_3$, 400 MHz) δ = 1.29 (3H, t, *J* = 7.0 Hz, -$COCH_2CH_3$), 1.45 (9H, s, -^{t}Bu), 1.55 – 1.66 (3H, m, -$NCH_2CH_2CH_2CH_2$NHBoc), 1.69 – 1.79 (2H, m, -$NCH_2CH_2CH_2CH_2$NHBoc), 3.18 (2H, q, *J* = 6.5 Hz, -$NCH_2CH_2CH_2CH_2$NHBoc), 3.94 (2H, t, *J* = 7.0 Hz, -$NCH_2CH_2CH_2CH_2$NHBoc), 4.22 (2H, q, *J* = 7.0 Hz, -$COCH_2CH_3$), 4.60 (1H, br. s, BocN*H*-), 6.96 (1H, d, *J* = 15.5 Hz, -NCOC*H*=CHCO-),7.17 (1H, d, *J* = 15.5 Hz, -NCOCH=C*H*CO-), 7.56 (2H, t, *J* = 8.0 Hz, *Ar*), 7.70 – 7.76 (1H, m, *Ar*), 8.11 – 8.15 (2H, m, *Ar*) ppm. ^{13}C NMR (101 MHz, Chloroform-*d*) δ 14.2, 27.1, 27.5, 28.5, 61.4, 79.4, 129.0, 130.3, 131.1, 133.5, 134.9, 156.1, 160.1, 165.3, 171.5. **FT-IR** ν_{max} (film) 3369, 2977, 2934, 1768, 1716, 1668, 1517, 1301, 1240, 1174, 1035, 1006, 708 cm^{-1}. **HRMS** (ESI-TOF) calcd for $C_{22}H_{30}N_2O_7Na$ $[M+Na]^+$: 457.1945, found: 457.1939.

Ethyl (*E*)-4-((4-aminobutyl)(benzoyloxy)amino)-4-oxobut-2-enoate (4)

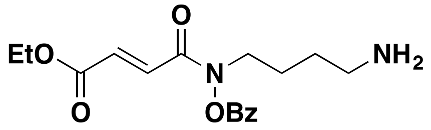

To a solution of (**3**) (2.05 g, 4.72 mmol) in CH_2Cl_2 (50 mL), was added trifluoroacetic acid (6.41 mL, 94.4 mmol) dropwise. The resulting mixture was allowed to stir at room temperature or 1 hour (TLC, EtOAc). The reaction mixture was then dried under *vacuo* before distilled water (50 mL) and CH_2Cl_2 (50 mL) was added. The organic material extracted with CH_2Cl_2 (2 × 50 ml). The organic layers were combined, dried over Na_2SO_4 , filtered and concentrated to yield the desired amine (**4**) as a pale yellow oil (1.50 g, 4.48 mmol, 95%) which was used without further purification. **^{1}H NMR** ($CDCl_3$, 400 MHz) δ = 1.22 (3H, t, *J* = 7.0 Hz, -$COCH_2CH_3$), 1.71 – 1.89 (4H, m, -$NCH_2CH_2CH_2CH_2NH_2$), 3.00 – 3.15 (2H, m, -$NCH_2CH_2CH_2CH_2NH_2$), 3.91 (2H, t, *J* = 6.0 Hz, -$NCH_2CH_2CH_2CH_2NH_2$), 4.16 (2H, q, *J* = 7.0 Hz, -$COCH_2CH_3$), 6.84 (1H, d, *J* = 15.5 Hz, -NCOC*H*=CHCO-), 7.10 (1H, d, *J* = 15.5 Hz, -NCOCH=C*H*CO-), 7.49 – 7.56 (2H, m, *Ar*), 7.66 – 7.77 (3H, m, *Ar*), 8.04 – 8.10 (2H, m, *Ar*) ppm. **^{13}C NMR** (101 MHz, $CDCl_3$) δ = 13.9, 23.9, 24.4, 39.7, 53.4, 61.5, 125.6, 129.1, 130.2, 133.8, 135.1, 161.0, 161.3, 165.0 ppm. **FT-IR** ν_{max} (film) 2980, 1768, 1719, 1669, 1452, 1394, 1201, 1177, 1139, 710 cm^{-1}. **HRMS** (ESI-TOF) calcd for $C_{17}H_{23}N_2O_5$ $[M+H]^+$: 335.1602, found: 335.1601.

(*E*)-4-(*N*-(Benzoyloxy)-4-ethoxy-4-oxobut-2-enamido)-*N,N,N*-trimethylbutan-1-aminium (5)

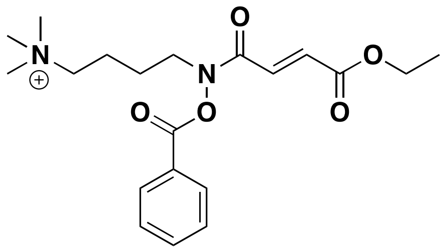

To a solution of (**4**) (1.50 g, 4.48 mmol) in dry acetonitrile (50 mL), methyliodide (1.76 mL, 28.3 mmol) and *N*,*N*-diisopropylethylamine (4.21 mL, 23.6 mmol) were added. The reaction mixture was stirred until consumption of starting material as observed by LCMS. The reaction mixture was concentrated, then distilled water (50 mL) and Et_2O (50 mL) were added. The aqueous phase was washed with Et_2O (5 × Et_2O), then frozen using liquid nitrogen, and lyophilised. The resulting crude mixture was purified by reverse-phase, flash column

chromatography using a 50 g Biotage® SNAP Ultra C18 column (1:99 to 0:100 MeCN(+0.1% TFA) /H_2O(+0.1% TFA) with the collection wavelength set to λ = 270 nm). Lyophilisation of the appropriate fractions gave quaternary amine (**5**) as a yellow oil (1.01 g, 2.69 mmol, 60%). **^{1}H NMR** (D_2O, 400 MHz) δ = 0.94 (3H, s, -$COCH_2C\mathit{H_3}$), 1.57 – 1.66 (2H, m, -$NCH_2CH_2C\mathit{H_2}CH_2NMe_3^+$), 1.69 – 1.78 (2H, m, -$NCH_2C\mathit{H_2}CH_2CH_2NMe_3^+$), 2.97 (9H, s, -N*Me$_3$*$^+$), 3.16 – 3.24 (2H, m, -$NC\mathit{H_2}CH_2CH_2CH_2NMe_3^+$), 3.76 – 3.94 (4H, m, -$COC\mathit{H_2}CH_3$, -$NCH_2CH_2CH_2C\mathit{H_2}NMe_3^+$), 6.60 (1H, d, *J* = 15.5 Hz, -NCOC*H*=CHCO-), 6.94 (1H, d, *J* = 15.5 Hz, -NCOCH=C*H*CO-), 7.38 (2H, t, *J* = 7.5 Hz, *Ar*), 7.57 (1H, t, *J* = 7.5 Hz, *Ar*), 7.90 (2H, d, *J* = 7.5 Hz, *Ar*) ppm. **^{13}C NMR** (101 MHz, D_2O) δ = 13.1, 19.6, 23.0, 47.7, 52.7, 62.0, 65.7, 129.1, 129.9, 130.3, 133.1, 135.4, 162.2, 162.5, 162.9 ppm. **FT-IR** ν_{max} (film) 2972, 1685, 1480, 1291, 1186, 1131, 720, 708 cm^{-1}. **HRMS** (ESI-TOF) calcd for $C_{20}H_{29}N_2O_5^+$ [M]$^+$: 377.2071, found: 377.2071.

(*E*)-4-(3-Carboxy-N-hydroxyacrylamido)-*N,N,N*-trimethylbutan-1-aminium (RL190B) (6)

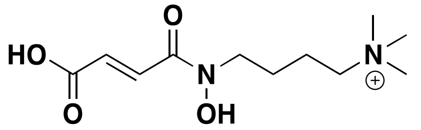

To a solution of (**5**) (500 mg, 1.33 mmol) in THF (13.3 mL) was added 1.0M $NaOH_{(aq)}$ (4.0 mL). The resulting mixture was stirred at room temperature for 3 hours until complete removal of both benzoyl and ethyl ester moieties was observed by LCMS. The reaction mixture was concentrated in *vacuo*, then lyophilised and purified by reverse phase flash column chromatography using a 50 g Biotage® SNAP Ultra C18 column (1:99 to 0:100 MeCN(+0.1% TFA) /H_2O(+0.1% TFA). Fractions containing the product were assayed by TLC and LCMS before being collected and lyophilised to yield **6** a pale yellow oil (293 mg, 1.12 mmol, 74%). **^{1}H NMR** (DMSO-*d*$_6$, 400 MHz) δ = 1.54 – 1.63 (2H, m, -$NCH_2C\mathit{H_2}CH_2CH_2NMe_3^+$), 1.63 – 1.74 (2H, m, -$NCH_2CH_2C\mathit{H_2}CH_2NMe_3^+$), 3.03 (9H, s, -$NMe_3^+$), 3.26 – 3.33 (2H, m, -$NC\mathit{H_2}CH_2CH_2CH_2NMe_3^+$), 3.64 (2H, t, *J* = 7.0 Hz, -$NCH_2CH_2CH_2C\mathit{H_2}NMe_3^+$), 6.56 (1H, d, *J* = 15.5 Hz, -NCOC*H*=CHCO-), 7.47 (1H, d, *J* = 15.5 Hz, -NCOCH=C*H*CO-). **^{13}C NMR** (101 MHz, DMSO-*d*$_6$) δ = 19.4, 23.1, 39.9, 46.9, 52.2, 64.9, 131.4, 132.7, 163.5, 166.4. **FT-IR** ν_{max} (film) 3437, 1659, 1436, 1407, 1311, 1020, 952, 932, 698, 668 cm^{-1}. **HRMS** (ESI-TOF) calcd for $C_{11}H_{21}N_2O_4^+$ [M]$^+$: 245.1496, found: 245.1496.

References

1 A. M. Rydzik, I. K. H. Leung, G. T. Kochan, N. D. Loik, L. Henry, M. A. McDonough, T. D. W. Claridge and C. J. Schofield, *Org. Biomol. Chem.*, 2014, **12**, 6354–6358.

2 A. M. Rydzik, J. Brem, S. S. van Berkel, I. Pfeffer, A. Makena, T. D. Claridge and C. J. Schofield, *Angew Chem Int Ed Engl*, 2014, **53**, 3129–3133.

3 F. H. Niesen, H. Berglund and M. Vedadi, *Nat. Protoc.*, 2007, **2**, 2212–2221.
